# Children as complex patients

**DOI:** 10.3325/cmj.2024.65.70

**Published:** 2024-02

**Authors:** Aleksandar Džakula, Iva Lukačević, Lovrenčić, Aida Mujkić

**Affiliations:** 1 Department of Social Medicine and Organization of Health Care, Andrija Štampar School of Public Health, University of Zagreb School of Medicine, Zagreb, Croatia; *iva.lukacevic.lovrencic@snz.hr*

Children are our greatest treasure, our future, and a source of happiness. But what if the child faces a severe illness or great suffering? Is it just a medical phenomenon, or is it, in itself, a complex challenge?

A child's illness is a challenge from many perspectives. Children are dependent on their parents, caregivers, and people from the immediate vicinity, and this is why we always see a child's illness as an illness of a small community. A child's disease is not a disease of a small person or a small adult. By definition, a child is a person undergoing biological, psychological, and social development. Children's health care must be adequate, precise, and timely. Only as such, can it ensure conditions for normal growth and development. This is why a disease that threatens the life of a child or a chronic illness that will follow a young person for the rest of their life is a unique challenge.

However, from the position of those responsible for the organization of care, the most significant challenge is caring for the health of children with complex needs (1-3). Such complex conditions do not have to be particularly severe. Still, in combination with several diseases, social problems, or severe mental conditions, they represent the biggest challenge for everyone - parents, health care, school, and community. While the medical part of complexity can be relatively easily introduced into the framework of modern medicine and treatment options, we must adapt all other parameters and determinants of care to immediate and specific local conditions (3-5). In such a situation, even the most potent medical technology remains trapped by personal and social circumstances.

## Family and parents

The family represents the immediate medium in which the child grows and develops, and as such, is often the source of problems, but also of solutions. The traditional family coexists with modern family structures, especially in today's global circumstances characterized by migration, wars, climate change, and economic and political turmoil. In this context, children are in multiple ways affected by the social determinants of health - those related to the family, the local community, and society as a whole.

The parent of a child with complex needs takes on multiple roles - apart from raising, guiding, supporting, and loving the child, he or she also coordinates care. In practice, the parent investigates rights and obligations, fights with or against the system, advocates, and lobbies. Also, the parent often sacrifices his or her health, work status, and financial security to meet the child’s needs (6,7). Parental responsibility and care for the child is expected, implied by everyone, and prescribed by law (8,9). However, the relationship of the system toward the parent is often vague, inconsistent, unequal in practice, and frustrating for both experts and parents (10).

## The health care system

Regardless of whether we experience health problems, our lives in modern society have been linked to the health system from the very beginning. We are born in health institutions, and throughout our childhood, we go through all the necessary examinations or procedures that should enable us to live a long and healthy life. From this, it could be concluded that medicine and health care are entirely and precisely informed about children's problems, and that it is difficult to expect any significant deviations or difficulties in care.

However, modern medicine and health care development have not been focused on a complete understanding of children's problems, but rather on narrow biomedical aspects related to certain medical phenomena. In such circumstances, numerous silos of diagnosis and therapy have been created, within which very successful and powerful medical interventions take place. But between them lies a space in which we do not respond to the entirety of the child’s needs.

This deviation from the concept of comprehensive care can, in most cases, be compensated in different ways by other parts of health or social care. Parents, non-governmental organizations, and local communities often bridge these gaps. However, in the case of children with complex needs, this lack of integration is not just a marginal phenomenon but becomes an essential problem that limits a good outcome, satisfactory quality, and conditions for a child's normal life (3).

This is why it is imperative that the health system, together with other public services, such as social care and education, creates a complete network in which we will be able to catch all the problems and needs of children with complex needs. It should form a comprehensive network of integrated care, together with parents and based on such recognized needs (1,3,11).

The problem of fragmentation arises in more than just the health system or other institutions that care for children. Its roots are in modern society, which is moving in the direction of narrow specializations in all fields. Then, this misunderstanding of needs comes back to us in the form of poor care for children. This is why interventions are necessary in institutions for the education of future professionals. Namely, schools and colleges that prepare experts for future work with children must train future experts to recognize the complexity of needs, avoid fragmentation, and see their future work not only as a personal professional challenge but as an invitation to team up with other professionals and parents to create comprehensive care for children (11).

## Society and the state

Normatively, children are extensively and multidimensionally protected both at the international and national level. For example, the Republic of Croatia is a signatory to the Convention on the Rights of the Child and the European Convention on the Exercise of Children's Rights. The European Union adopted the Strategy on the Rights of the Child, an integral part of which is the European Child Guarantee (12-15). Croatia has an independent institution of the Ombudsman for Children and the Council for Children at the Ministry of Labour, Pension System, Family, and Social Policy. It also formulated the National Plan for Children's Rights in the Republic of Croatia for the period from 2022 to 2026, and the National Plan of Equalization of Opportunities for Persons with Disabilities for the period from 2021 to 2027 (16,17). Children are also included in the current national plans for the development of health care, combating poverty and social exclusion, the development of social services, and Roma inclusion.

In a top-down translation into a real context, this extensive and dense network of legislative and strategic support for children is becoming less and less dense. The Croatian public regularly hears about children and families who “fall through the cracks” of the system, with unsatisfied rights and without clearly structured and systematic support.

Children with complex needs require interventions and resources readily available in real-life contexts. These resources must be in a system that is stable, resistant, firmly structured, organized, efficient, and effective, but also flexible, accessible, fair, and equitable. The goal is not only to provide humanitarian aid but also to achieve the highest level of health in the broadest possible scope, and what is especially important, not to create an unbearable burden for the family.

Generally speaking, today's system of care for children with complex needs lacks integration at the systemic level and care coordination at the individual level. The existing systems offer the child and the family an episodic service, burdened with the struggle to achieve normatively guaranteed rights and without an actual response to the child's and the family's need for continuity of care and systematic support. Various “bottom-up” proposals for improvement mainly come from the activism of parents supported by non-governmental organizations. In this way, they respond to the lack of resources and the translation of the strategic vision into actual steps in the public sector. However, such initiatives are difficult to reach the decision-makers, and a lot of advocacy by parents, non-governmental organizations, and professionals is needed for decisions to be made at the top (10). Just as it is difficult for existing forms and resources to reach children with complex needs, it is also difficult for initiatives for improving care to reach decision-makers. Ultimately, both children and their care share the same fate: insufficient understanding, lack of integration, and underestimation of complexity!

## References

[1] Kuo DZ McAllister JW Rossignol L Turchi RM Stille CJ Care coordination for children with medical complexity: whose care is it, anyway? Pediatrics 2018 141 Suppl 3 S224 32 10.1542/peds.2017-1284G 29496973

[2] Brenner M Greene J Doyle C Koletzko B del Torso S Bambir I Increasing the focus on children’s complex and integrated care needs: a position paper of the European Academy of Pediatrics. Front Pediatr 2021 9 10.3389/fped.2021.758415 34926344 PMC8671931

[3] Brenner M, Alma M, Clancy A, Larkin P, Lignou S, Luzi D, et al. Models of child health appraised. work package 2: report on needs and future visions for care of children with complex conditions. Brussels: European Commission, 2017. Available from: https://www.childhealthservicemodels.eu/wp-content/uploads/20171130_Deliverable-D11-2.4-Report-on-needs-and-future-visions-for-care-of-children-with-complex-conditions.pdf. Accessed: December 13, 2023.

[4] Cassidy L Quirke MB Alexander D Greene J Hill K Connolly M Integrated care for children living with complex care needs: an evolutionary concept analysis. Eur J Pediatr 2023 182 1517 32 10.1007/s00431-023-04851-2 36780041 PMC9924191

[5] Council on Children with Disabilities and Medical Home Implementation Project Advisory Committee Patient- and family-centered care coordination: a framework for integrating care for children and youth across multiple systems. Pediatrics 2014 133 e1451 60 10.1542/peds.2014-0318 24777209

[6] Banadinović M Vočanec D Lukačević Lovrenčić I Lončarek K Džakula A Role and perspectives of informal care: a qualitative study of informal caregivers in the Republic of Croatia. BMJ Open 2023 13 e074454 10.1136/bmjopen-2023-074454 37827736 PMC10582946

[7] Leung BM Wandler C Pringsheim T Santana MJ Working with parents of children with complex mental health issues to improve care: A qualitative inquiry. J Child Health Care 2022 26 548 67 10.1177/13674935211028694 34180250 PMC9667073

[8] Ministry of Labour, Pension System, Family and Social Policy of the Republic of Croatia. Law on Social Welfare. Zagreb: Ministry of Labour, Pension System, Family and Social Policy of the Republic of Croatia, 2022. Available from: https://www.zakon.hr/z/222/Zakon-o-socijalnoj-skrbi. Accessed: January 24, 2024.

[9] Ministry of Labour, Pension System, Family and Social Policy of the Republic of Croatia. The Family Law. Zagreb: Ministry of Labour, Pension System, Family and Social Policy of the Republic of Croatia, 2023. Available from: https://www.zakon.hr/z/88/Obiteljski-zakon. Accessed: January 24, 2024.

[10] Lukačević Lovrenčić I Banadinović M Mujkić A Džakula A Djeca s kompleksnim potrebama u Republici Hrvatskoj – okolnosti i mogućnosti za unaprjeđenje skrbi. Medix. 2022 155 54 8

[11] Kuo DZ Houtrow AJ Council on children with disabilities. recognition and management of medical complexity. Pediatrics 2016 138 e20163021 10.1542/peds.2016-3021 27940731

[12] United Nations. Convention on the Rights of the Child. Geneva: United Nations, 1989. Available from: https://www.ohchr.org/en/instruments-mechanisms/instruments/convention-rights-child. Accessed: January 5, 2024.

[13] Council of Europe. European Convention on the Exercise of Children's Rights. Strasbourg: Council of Europe, 1996. Available from: https://rm.coe.int/european-convention-on-the-exercise-of-children-s-rights/1680a40f72. Accessed: January 5, 2024.

[14] European Commission. EU strategy on the rights of the child. [Internet] Brussels: European Commission, 2021. Available from: https://eur-lex.europa.eu/legal-content/en/TXT/?uri=CELEX%3A52021DC0142. Accessed: January 5, 2024.

[15] The Council of the European Union. European Child Guarantee. [Internet] Luxembourg: The Council of the European Union, 2021. Available from: https://eur-lex.europa.eu/legal-content/EN/TXT/?uri=uriserv%3AOJ.L_.2021.223.01.0014.01.ENG&toc=OJ%3AL%3A2021%3A223%3ATOC. Accessed: January 5, 2024.

[16] Ministry of Labour, Pension System, Family and Social Policy of the Republic of Croatia. National Plan for Children's Rights in the Republic of Croatia for the period from 2022 to 2026. Available from: https://mrosp.gov.hr/UserDocsImages/dokumenti/Socijalna%20politika/Dokumenti/Nacionalni%20plan%20za%20prava%20djece%20u%20Republici%20Hrvatskoj%20za%20razdoblje%20od%202022.%20do%202026.%20godine.pdf*.*Accessed: January 15, 2024.

[17] Ministry of Labour, Pension System, Family and Social Policy of the Republic of Croatia. National Plan of Equalization of Opportunities for Persons with Disabilities for the period from 2021 to 2027. Available from: https://mrosp.gov.hr/UserDocsImages/dokumenti/Glavno%20tajni *štvo/Godišnji%20planovi%20i%20strateška%20izvješća/Nacionalni%20plan%20izjednačavanja%20mogućnosti%20za%20osobe%20s%20invaliditetom%20za%20razdoblje%20od%202021%20do%202027.%20godine.pdf*. Accessed: January 15, 2024.

